# Time- and Resource-Efficient Time-to-Collision Forecasting for Indoor Pedestrian Obstacles Avoidance

**DOI:** 10.3390/jimaging7040061

**Published:** 2021-03-25

**Authors:** David Urban, Alice Caplier

**Affiliations:** 1 CNRS, GIPSA-Lab, Institute of Engineering, University of Grenoble Alpes, 38000 Grenoble, France; david.urban@gipsa-lab.grenoble-inp.fr or; 2 IKOS RA, 69001 Lyon, France

**Keywords:** deep learning, collision detection, Time-to-Collision prediction, real-time, object detection, monocular depth estimation

## Abstract

As difficult vision-based tasks like object detection and monocular depth estimation are making their way in real-time applications and as more light weighted solutions for autonomous vehicles navigation systems are emerging, obstacle detection and collision prediction are two very challenging tasks for small embedded devices like drones. We propose a novel light weighted and time-efficient vision-based solution to predict Time-to-Collision from a monocular video camera embedded in a smartglasses device as a module of a navigation system for visually impaired pedestrians. It consists of two modules: a static data extractor made of a convolutional neural network to predict the obstacle position and distance and a dynamic data extractor that stacks the obstacle data from multiple frames and predicts the Time-to-Collision with a simple fully connected neural network. This paper focuses on the Time-to-Collision network’s ability to adapt to new sceneries with different types of obstacles with supervised learning.

## 1. Introduction

### 1.1. Technical Context

Real-time computer vision-based scene understanding tasks can be very complex problems especially for navigational systems like autonomous driving and drone flying as the onboard camera is moving asynchronously to its environment and the system needs to adapt to new paths, sceneries, and obstacles. Less popular than these types of systems discussed in recent the literature, the pedestrian egocentric navigation system has very similar problems [1]. As a matter of fact, many vision-based embedded system prototypes using multiple sensors and cameras exist for the visually impaired and blind involving heavy portable GPU equipment and sensors [2,3]. The considered industrial application is an interactive pedestrian guiding system mounted on smartglasses for the visually impaired. The proposed navigation system is built around the hypothesis that it can function on a smartphone or smartglasses device (like Google Glass or Epson Moverio BT300) using only their built-in sensors (video camera, internal IMU: Inertial Measurement Unit, and GPS: Global Positioning System). In order to provide guiding indications to the user, the system must process the embedded camera video flow data as fast as possible considering the size of the device. In this paper, we focus on the task of Time-to-Collison prediction while limiting the input sensor to a smartglasses’ camera point of view only. As the user of the system may encounter various types of incoming obstacles on his/her path, the objective of this study is to understand how challenging collision detection can be without specifying the type of encountered obstacles (pedestrians, vehicles, poles, trash cans, etc.). The solution needs to be efficient enough to work on real-world scenes and simple enough to run on a smart device in a real-time application. The proposed method has to detect the obstacles, on the user’s path and to predict the time until the obstacle reaches the user or until the distance between the user and the obstacle is less than one meter.

In this work, we propose a model that extracts spatial features from the egocentric pedestrian video stream in a static data extractor module such as the scene’s depth maps and the obstacles’ location from state-of-the-art light weighted algorithms. These features stacked on multiple frames are fed in a dynamic data extractor that introduces our novel obstacle Time-to-Collision prediction neural network. We present the different datasets used for training and testing the new network. We present experiments on the network hyperparameters choices in order to balance execution robustness and complexity. Results on both mobile and static obstacles on an untrained dataset are presented to demonstrate the solution’s ability to generalize on a new environment. We compare our results with the state-of-the-art solution on the available dataset for this task.

### 1.2. Related Work

Collision detection algorithms are trending in the field of autonomous driving for cars or drones [4,5]. Usual vision-based navigation system solutions are based on multiple cameras and sensors from the vehicle with both the video color camera and a type of depth camera (stereo, or LIDAR [4]) as the depth scene analysis is the best way to localize and predict the obstacle relative movements to the vehicle. However, with the case of small drones [5] or a navigation system for smartglasses, the device’s need to be light weighted forces the input data to be restricted to only a monocular video camera. Evidently, it is a difficult task to evaluate a collision risk without the scene’s depth data.

One way to avoid using the depth data as input is to use structure from motion methods like optical and scene flow reconstruction [6,7]. The problem with these methods is that they are computationally heavy dealing with multiple frames at the same time. In addition, optical flow algorithms are less effective for obstacles moving toward the camera because of the aperture problem. The monocular depth estimation task is an equally difficult problem. However, the depth map can be generated from still image information instead of motion cues which reduces the complexity consequently. The state-of-the-art solutions use deep learning convolutional neural networks [8,9].

An intuitive solution [5,10] to obstacle avoidance or collision detection is to separate the task in two subtasks: an obstacle detection algorithm followed by a risk estimator or a direction selector. Real-time effective state-of-the-art object detection based on neural networks [11,12] can easily find specific obstacles when properly trained [10,13]. A few datasets for autonomous car driving tasks contain context appropriate annotations for outdoor pedestrian obstacle detection [14,15]. The risk estimator is usually tackled with the “relative distance” from the camera focal and knowledge of obstacle width, or the “relative velocity” [5,6,10]. These methods use temporal cues and rely on a few frames in order to predict a possible risk. The authors of [5] presented a method that detects the SIFT (Scale-Invariant Feature Transform) feature points of the obstacles and decides if the detected obstacle may cause a collision by comparing the area ratio of the obstacle and the position of the drone. The authors of [10] presented a method that detects the vehicles with Faster-RCNN [11] and uses the bounding box coordinates with the camera intrinsic parameters to compute the relative distance and velocity. The authors of [13] have a similar concept but use a small fully connected neural network to evaluate the distance from the bounding boxes. The authors of [6] and [10] both use Time-to-Collision as a risk factor computed with the relative velocity between the camera and the obstacle to confront.

The Time-to-Collision (or Time-to-Contact [16]) prediction is the most suitable risk estimator for our smartglasses navigation system instead of obstacle or relative speed estimation [17]. However, this computation is often derived from the scene’s motion flow [18,19,20,21]. Segmentation or feature-based approaches [22,23,24] are also used to compute Time-to-Collision as an optical flow problem. It can be done by computing the scaled expansion of the segmented regions or the matching features of the obstacles on multiple frames. An interesting end-to-end Deep Learning method, called Near-Collision in [25], skips the obstacle detection task and the relative motion computation to estimate directly the Time-to-Collision with incoming pedestrians. Despite the network being fast and robust, estimating only the time to collision without the obstacle characteristics is not enough for a guiding system with too many types of possible obstacles. We propose a novel method to address the Time-to-Collision problem on a monocular navigation system by using only light weighted neural networks.

## 2. Proposed Model

### 2.1. Obstacle Detection Algorithm for the SmartGlasses Navigation System Application

**Figure 1:**
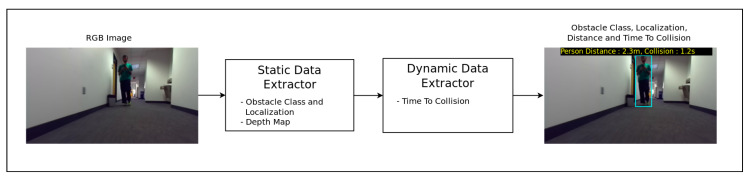
Smartglasses navigation system obstacle detection pipeline.

The model comprises two modules (Figure 1):

- A static data extractor that computes the scene depth map and identifies and localizes the presence of an obstacle;
- A dynamic data extractor that uses temporal cues from the data computed in the first module stacked along multiple frames and predicts the Time-to-Collision to the closest obstacle detected.

This article focuses mainly on the proposed method and evaluation results of the second module.

#### 2.1.1. Static Data Extractor

**Figure 2:**
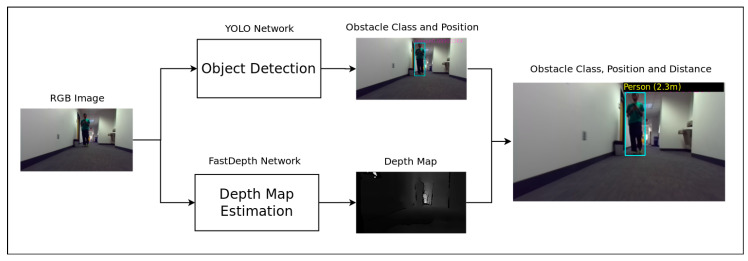
Smartglasses navigation system static data extractor module. The depth map is a grayscale image where the brightest pixels represent the furthest distance and the darkest pixels the closest one. After estimating the bounding box and the depth map, each obstacle distance to the user is computed as the minimum depth value estimated within each bounding box applied on the depth map.

This module (Figure 2) exploits spatial features from each color video frame in order to help identify and localize the user’s environment. It computes from the monocular color video the obstacles position (bounding box coordinates and dimensions) and the depth map. The localization and object recognition tasks are performed by a retrained version of the YOLOv3 [12] real-time object detection neural network. The network, initially designed and trained in order to recognize 20 classes, was retrained from the Darknet-53 backbone on the COCO [26] and SUN-RGB [27] datasets to detect only 3 classes to limit the task to more context appropriate outputs:

- “Person”: this class represents the mobile obstacles the smartglasses user would encounter (pedestrians, vehicles).
- “Chair”, “desk”: these classes illustrate some examples of “static” obstacles the smartglasses user would have to avoid on his/her path.

Those classes were selected according to the data available in existing accessible datasets and the testing environment we have chosen to test our solution (indoor office).

The depth map estimation is performed by the lightweight robust real-time FastDepth neural network proposed in [8].

**Figure 3:**
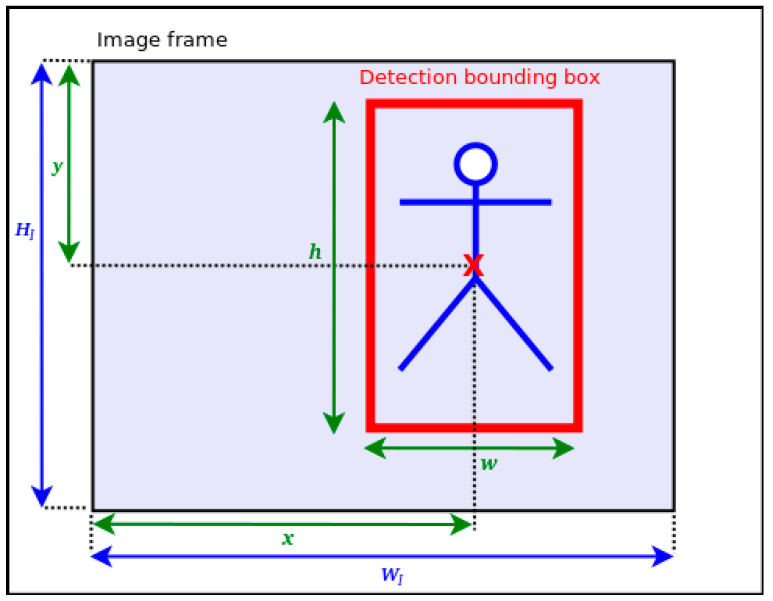
Description of a detected bounding box (BBox) on a frame. *x* and *y* are the BBox center coordinates. *h* and *w* denote, respectively, the BBox’s height and width. ($W_{I},H_{I})$ are the image dimensions.

From the data computed by the static data extractor, we select five compact pieces of information (cf. Figure 3) which are the coordinates *x* and *y* of the center of each object bounding box, *w* and *h* its dimensions and *D* the minimum depth value estimated within each bounding box. For the purpose of Time-to-Collision estimation, some data are normalized as follows:

(1) $$X=x/W_{I}\;Y=y/H_{I}\;W=w/W_{I}\;H=h/H_{I}$$

#### 2.1.2. Dynamic Data Extractor

**Figure 4:**
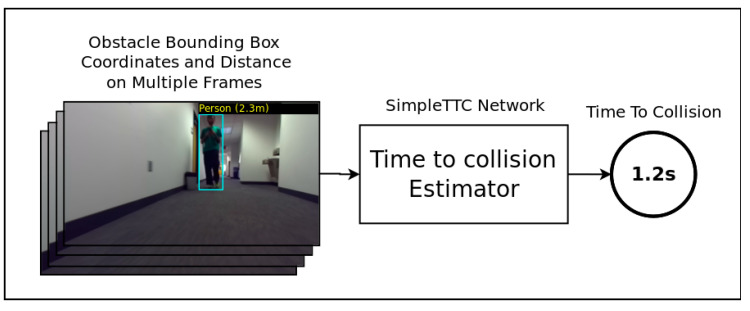
Smartglasses navigation system dynamic data extractor module.

The dynamic data extractor (Figure 4) exploits temporal features contained in the data extracted from multiple frames passed through the first module. This module is used as a predictive evaluation of the user’s environment to allow the navigation system to anticipate the user’s possible interactions with the obstacles in his/her path. Instead of using the complete RGB frames from the video and using an elaborate convolutional neural network for temporal processing as in [28], we propose a very simple method by first limiting the input of our model to five values per frame (bounding box’s center point normalized coordinates *(X, Y)*, dimensions *(W, H),* and *D* the minimal distance value of the detected obstacle) and secondly by processing those data with a simple neural network. The system outputs the Time-to-Collision which is the time it will take for the user to reach or pass by the incoming obstacle.

**Figure 5:**
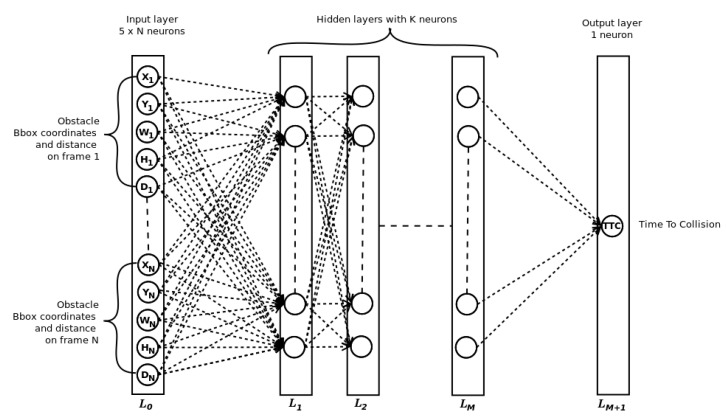
Simple TTC network architecture. In order to find the best setup, we experiment with the number of input frames *N*, the number of hidden layers *M* and the number of neurons per hidden layers *K*.

The proposed Time-to-Collision forecasting system is a fully connected deep neural network called SimpleTTC (Figure 5). The network architecture is made of *M* hidden layers with *K* neurons per hidden layer. The input layer takes the obstacle’s parsed data *X*, *Y W*, *H* and *D* stacked on *N* frames of the video. The prediction is established for the current frame of the sequence to predict the Time-To-Collision.

The network is trained with a 0.001 learning rate and a stochastic gradient descent optimizer for up to 100 epochs. The successive input frames are sampled at 10 fps which gives the input temporal window a history of (*N* − 1)/10 s.

The mean squared error is used as the loss function:

(2) $$\ell \left(y,y^{*}\right)\;=\;L=\;\mathrm{mean}(\{l_{1},\ldots ,\;l_{S}\}),\;l_{s}={\left(y_{s}-y_{s}^{*}\right)}^{2}$$

where *S* is the number of sequence samples. y and $y^{*}$ are, respectively, the predicted Time-to-Collision and the ground-truth.

### 2.2. Datasets

To train and evaluate the proposed network four pieces of information are needed:

- The RGB video sequence with the incoming obstacles in the view;
- The corresponding depth map extracted from either a stereo camera disparity map, the LIDAR point cloud coordinates, or the output of a monocular depth map estimator algorithm;
- The bounding box of the obstacles annotated either manually or with the help of a state-of-the-art detection algorithm;
- The Time-to-Collision value corresponding to each frame where the prediction should be (as we consider the Time-to-Collision task to be a regression problem).

**Figure 6:**
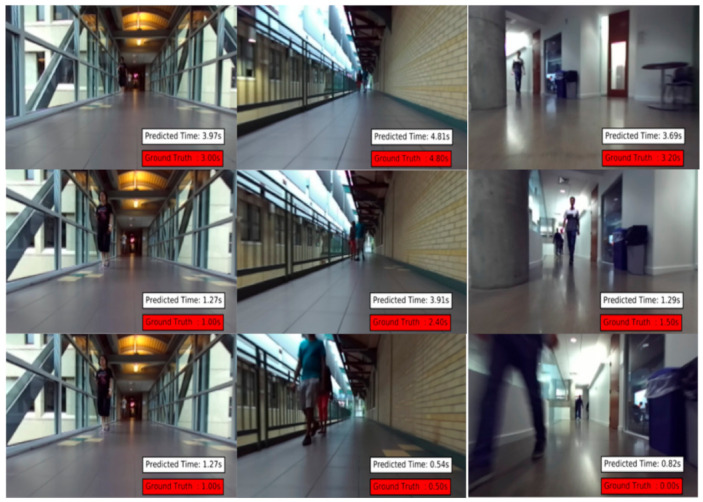
Three different test videos from the Near-Collision dataset with their labeled Time-to-Collision ground truth and their predicted time [25].

The dataset used for training and evaluation is the Near-Collision set [25] consisting of indoor hallways egocentric videos with time to collision annotations of incoming pedestrians (Figure 6). The videos were taken from an embedded camera mounted on a suitcase shaped prototype. The context of this setup is very similar to the one of the smartglasses navigation system. The dataset contains also the raw data used to annotate the Time-to-Collision which are the stereo images and the 3D point cloud data from a LIDAR. The bounding box data are generated from the Faster-RCNN person detection [11] and completed manually for missing data and false-positive. The Time-to-Collision is annotated from zero to six seconds. For each frame with an incoming pedestrian, a value is calculated as the time it takes for the pedestrian to be less than one meter away from the camera. The distance to the pedestrian is the minimal value of the depth map in the detected bounding box area. Because their official test split contains only the input sequence and the annotated Time-to-Collision but not the corresponding depth data, a new test split was regenerated from the raw data available. In fact, the whole dataset has been reformatted so that the bounding box and depth data could be used as input when labeling the Time-to-Collision. Ninety sequences of pedestrian near-collision cases are used in total for training and testing.

**Figure 7:**
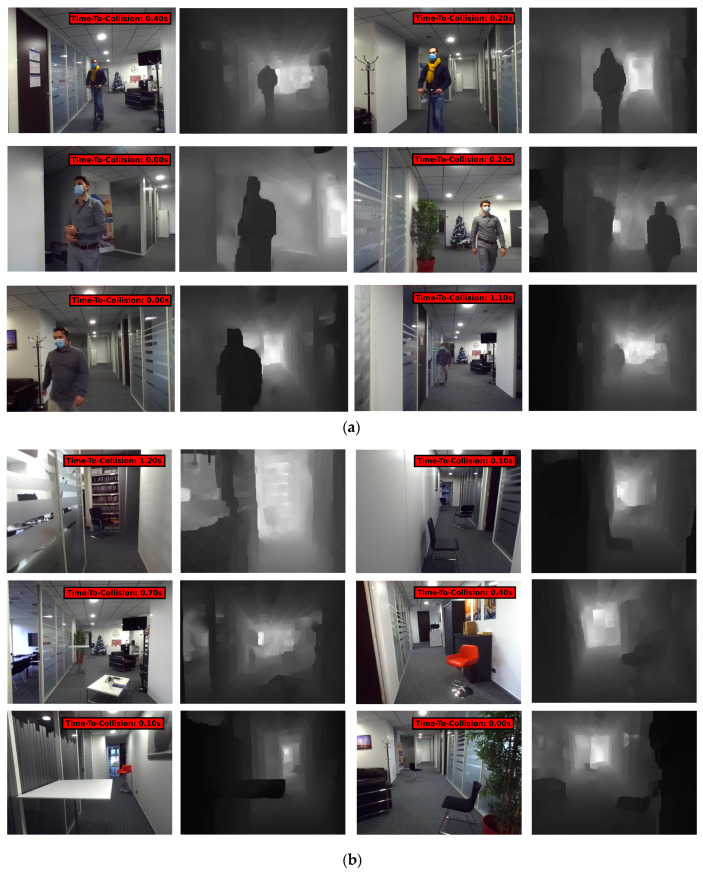
Our dataset used for testing purpose. Color RGB frames with labeled Time-to-Collision and corresponding depth maps. (**a**) Videos with only dynamic obstacles (pedestrians and scooter); (**b**) videos with only static obstacles on the path (chairs and desks).

A new dataset more suitable in the smartglasses navigation system context has been acquired (Figure 7). As a matter of fact, the Near-Collision dataset contains only mobile pedestrians as possible obstacles. No static obstacles are available. Note that this new dataset is used for testing purposes only. It contains similar images of indoor hallways videos. However, the path contains incoming pedestrians but also static obstacles like chairs and desks. These additional obstacle types simulate the variety of size, shape, and relative movement of the applicative real obstacle the system must detect and process. The images have been collected from an embedded stereo camera coupled with a NVIDIA Jetson TX1 development board. The videos were filmed while holding the camera at head level height to simulate the egocentric video from a camera embedded in smartglasses. This dataset contains the left-right stereo images with the associated depth maps computed directly by the ZED stereo camera API. More than 20 trips have been acquired with 17 instances of collision. Time-to-Collision labels were generated in the same way as in the case of the Near-Collision dataset that is to say “collision” is labeled when the obstacle is estimated to be less than one meter away from the user from the depth map (minimal value in the bounding box area). The detection algorithm used to recognize the obstacles and localize their bounding box is the retrained YOLOv3 network from the Static Data Extractor module that is able to use the “person”, “chair”, and “desk” classes as obstacles. This dataset is only used as a test set because of the limited number of acquired samples and also to be able to see if the SimpleTTC network can generalize the task from one dataset to another (dataset cross-validation) and from one type of obstacle to another (from mobile objects to static ones).

## 3. Experimental Results

### 3.1. Network Configuration

The architecture chosen for this task is a simple fully connected network (Figure 5). The network parameters to be considered in this experiment are the number of hidden layers *M* and the number of neurons *K* per hidden layer. The number of input frames is fixed in this particular experiment at six (the same as for the Near-Collision network [25]). The network is trained for 100 epochs with each configuration and the results in Table 1 are always the best out of 100 epochs. We compute the mean absolute error and the standard deviation for the test set of the Near Collision dataset to quantify the performance. As can be seen in Table 1, the best configuration with 6 input frames is 8 layers and 60 neurons per layer. The number of hidden layers matters. However, the number of neurons per hidden layer does not have a huge influence on the performance. For the remaining part for the paper, the chosen architecture is *M* = 8 and *K* = 60.

### 3.2. Number of Input Frames

In this experiment we compare the results for different numbers of frames (Table 2). As the lightest amount of processed data is prioritized for the model, it is important to find out how small the input of the network can be. The time history is (*N* ‒ 1)/10 s where *N* is the number of input frames. The results in Table 2 show that the model does not lose a significant amount of performance if we reduce the number of frames from six to three, therefore reducing the time window from 0.5 to 0.2 s and reducing by half the size of the input layer and the number of frames needed to predict the Time-to-Collision. For the remaining part of the paper the chosen value for the number of frames is *N* = 3.

The number of input frames used to predict the Time-to-Collision determines the time history of the filmed sequence. In the case of the Near-Collision dataset, the speed between the camera and the incoming pedestrians is relatively constant. This helped our network generalize the predictions for these particular situations in a small-time history (0.3 s). Nevertheless, if the speed changes a lot as in the case of a smartglasses user with possible pauses in his/her trip, the influence of the time history size should be more substantial. The results in Table 1 show that the depth of the network is more relevant than its thickness. The number of non-linear computations from the data acquired on multiple frames to make a linear regression prediction has yet a direct effect on the performance.

### 3.3. Time to Collision Estimation Results on the Near-Collision Test Set

**Figure 8:**
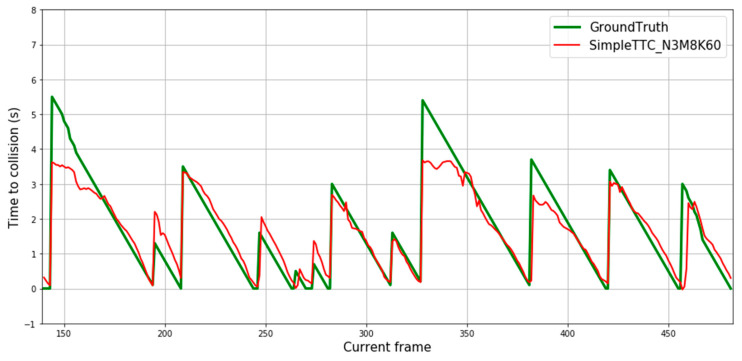
Prediction results over multiple frames in the Near-Collision test set. Each continuous descending line in the ground truth represents the same encounter with the obstacle until it leaves the view. Therefore, each figure’s slide represents one video sequence of “near-collision” with a different pedestrian.

Figure 8 depicts predictions of 12 different pedestrian “collision” encounters. Each spike of the ground truth’s curve represents the first frame of the sequence the prediction is made on. Predictions from our SimpleTTC model with 3 input frames, 8 hidden layers, and 60 neurons per layer show that the network is able to predict the Time-to-Collision consistently. However, the network struggles to predict correctly when the pedestrian is far from the camera (Time-to-Collision above 3 s).

### 3.4. Results on the Acquired Test Set

#### 3.4.1. Results on Moving Obstacles

**Figure 9:**
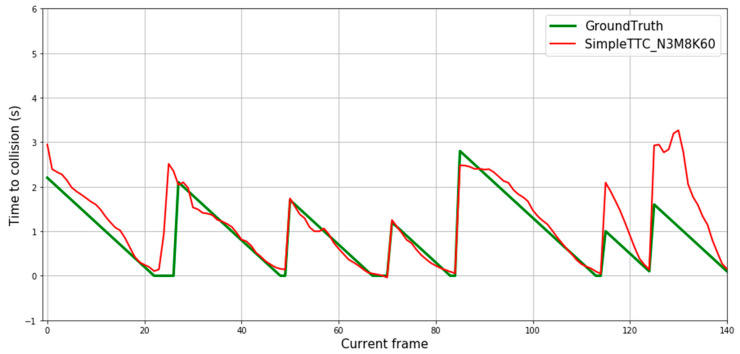
Prediction results over 3 multiple frames for our test dataset with moving obstacles. The SimpleTTC network while not trained on this dataset is able to perform accurate predictions of Time-to-Collision for incoming pedestrians. The last 3 sequences are collision encounters with the scooter.

Some prediction results of our generated dataset can be seen in Figure 9. The four first sequences are encountered with walking pedestrians. The next one is from a static position (the camera is not moving) with a scooter coming forward. The last two sequences are again from a walking point of view with the scooter coming forward. The goal of this experiment is to see if the solution can be cross-validated based on a very different dataset distribution with the same type of “near collision” events and also how it reacts to faster obstacles (a person on a scooter for instance). The relative speed is bigger in the last two sequences.

The SimpleTTC network predicts well on pedestrians, as the ground truth Time-to-Collision shape is reproduced by the prediction with small undesired fluctuations. For the predictions on the scooter with movement (last two slopes in Figure 9), the prediction slope starting point is twice higher than it should be. This is because the network assumes that the person on the scooter would take twice the time to reach the “collision” threshold and so on for all the following frames. If the network could differentiate between the different kinds of dynamic obstacles (walking pedestrian, running pedestrian, person on scooter, etc.), the Time-to-Collision output could be scaled to each average speed.

The SimpleTTC prediction makes accurate predictions for pedestrians within normal walking speed (cf. Figure 9). However, it is clearly unable to scale the prediction to match the speed change (encounters with the scooter) as it was not trained with this kind of data.

#### 3.4.2. Results on Static Obstacles

**Figure 10:**
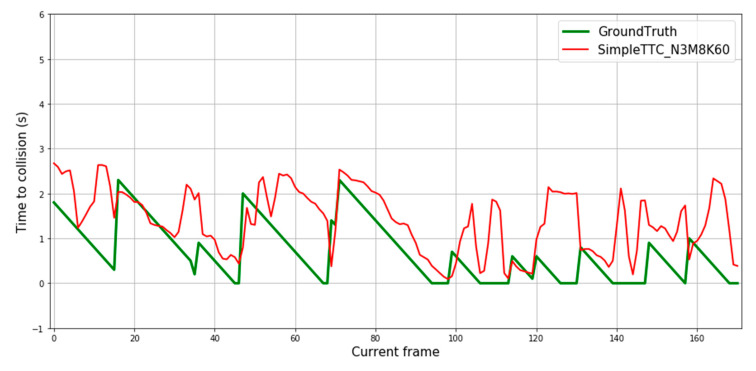
Prediction results over multiple frames for our dataset with static obstacles.

The second purpose of the proposed test set is to study the generalization of the trained model for different kinds of obstacles, more precisely for a static “chair” and “desk”. The obtained predictions for those static obstacles can be observed in Figure 10. For some collisions sequences the prediction seems to have a normal behavior with a slight detection gap (about 0.3 s) but the detection seems to fail most of the time.

The network is hardly able to correctly predict the Time-to-Collision of static objects. In the Near-Collision dataset, the pedestrians leave the frame from the left or right as the camera is placed at about one meter high. In our dataset the static obstacles leave the frame more in the bottom corner of the frame as the camera is leveled at eye height. This difference between the datasets and the fact that the bounding box size of static obstacles is smaller than that of pedestrians may confuse the network. It deduces that obstacles with smaller bounding boxes are further. The small gap between prediction and ground truth could also be explained by the smaller bounding box size and the different relative speed.

### 3.5. Computational Efficiency

The whole model (static + dynamic data extractor) runs smoothly at 25 fps on a Nvidia 1080Ti graphic card (NVIDIA, Santa Clara, CA, USA) and use 3297MiB of graphical memory (about 4 Gb). The solution is acceptable for the prototypal phase, as it needs to run on at least 10 fps for the model to function correctly.

The long-term goal is to have a lightweight robust model that could run on a smart device (smartphone, smartglasses). The GPU/CPU memory storage used by the system must be small enough to be able to process the real-time video flow and deliver with an under one second response time for this type of applications. Our model architecture is mostly optimized for this task in terms of input data and network size as we addressed the problem at hand as a simple regression problem.

### 3.6. Comparison with the Near-Collision Network

**Figure 11:**
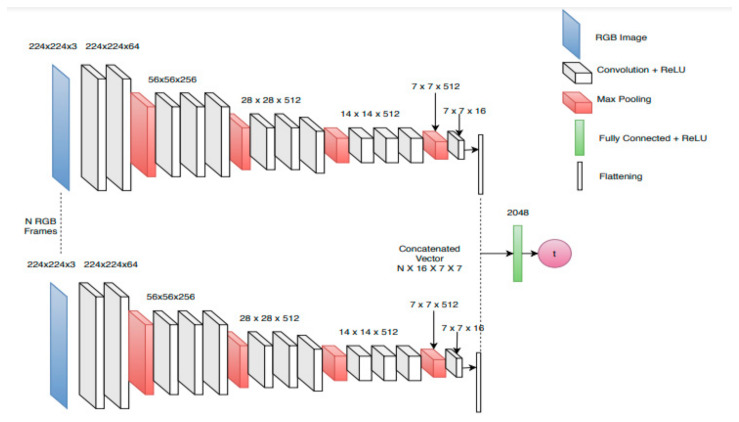
Near-Collision network architecture from [25]. It is a multi-stream that takes 6 consecutive RGB frames as input through the same convolutional network. At the end part of the network, the features from each stream are flattened and stacked together to process the data from all the frames in a last fully connected layer. The network outputs the Time-to-Collision. Reprinted with permission from ref. [25]. Copyright 2019 IEEE.

**Figure 12:**
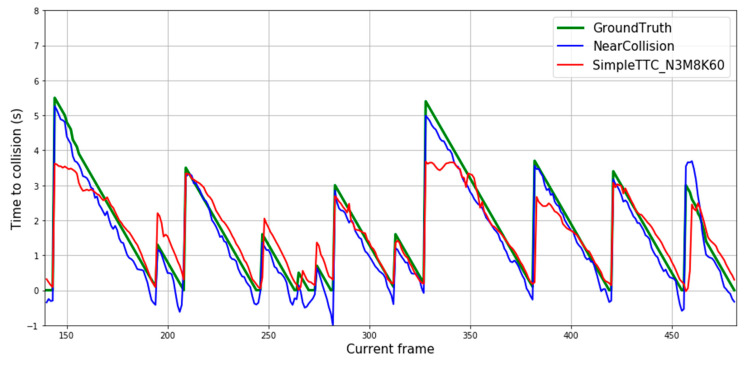
Prediction results over multiple frames in the Near-Collision dataset.

We compare our results with the Near-Collision method [25] as we tackle the Time-to-Collision problem in the same way. From the plot shown in Figure 12, predictions from our SimpleTTC network obtained with 3 input frames, 8 hidden layers, and 60 neurons per layer are compared to the Near-Collision network results (Figure 11) obtained with 6 input frames. For both counts the input data were sampled from the video frames at the same 10 fps frequency. Our method struggles more with predictions for further pedestrians (Time-to-Collision > 3 s), however, produces slightly smoother and more accurate results for closer pedestrians in the Near-Collision test set (Figure 12).

**Figure 13:**
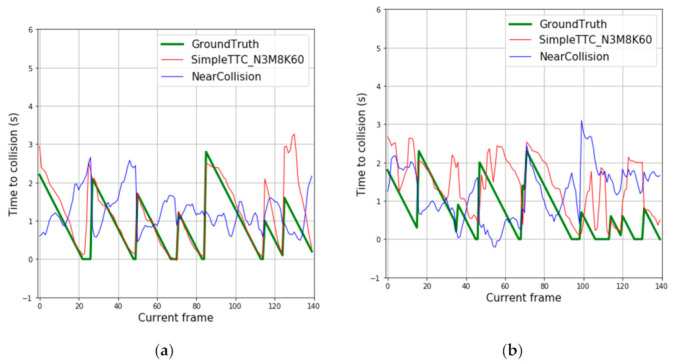
Predictions results based on our dataset for: (**a**) dynamic obstacles; (**b**) static obstacles.

However, their method does not generalize at all when dealing with our dataset (Figure 13) as the results of their prediction seem random and nonlinear.

The Near-Collision network [25] can accurately predict the Time-to-Collision on the Near-Collision dataset, but it does not generalize to our dataset (Figure 13). When restricting the data to the bounding box of the obstacles and their relative distance to the camera, our model generalizes the task well on a different dataset that does not even have the same camera height (about 0.8 m difference).

### 3.7. Comparison with an Improved Version of the NearCollision Network

**Figure 14:**
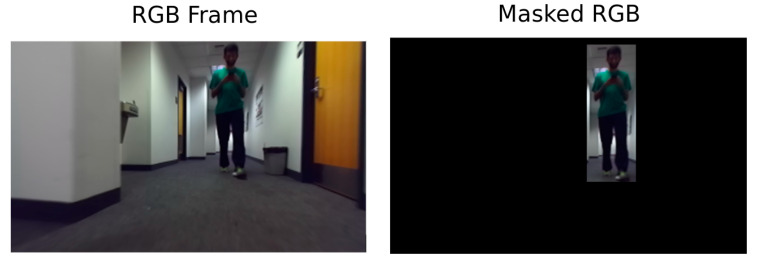
Example of input format for the Near-Collision network in our experiment. The “mask” is applied by setting the value of all pixels that are not within the obstacle bounding box to zero.

Initially the Near-Collision network is trained on the whole image. However, in the case where there are multiple objects in the same image, as it is the case when dealing with our dataset, it can introduce some noise in estimating the Time-to-Collision for a given object. That is why we conducted a new experiment to check how much better the Near-Collision architecture could improve while restricting the input layer to the bounding box around each obstacle (Figure 14). As the input data is gradually reduced, the efficiency of the features selected as input can be confirmed as improvement or not.

In this experiment we retrained the Near-Collision network by changing the input frames with different forms of the data outputs from the static data extractor module (Figure 14). The detected bounding box data was put at the input of a convolutional neural network (CNN) by applying a mask on the frame, removing the background of the obstacle. The architecture of the network remains unchanged. Each instance was retrained from VGG-16 [29] pre-trained weights. As can be seen in Table 3, restricting the input frame to the bounding box of the obstacle reduces the error by about 20%. However, our model is still about twice more accurate than the improved Near-Collision network.

This experiment shows that the position and shape of the obstacle’s bounding box and the depth information are the right features to exploit for Time-to-Collision forecasting. Finally, using only minimal depth and the bounding box shape of the obstacle in the SimpleTTC network removes the information about the obstacle 3D pose and orientation. However, the reduced input removes all the background image noise that would impede the training.

## 4. Conclusions

The SimpleTTC network is a 10-layer deep fully connected neural network that is fed with the obstacle’s location and depth data as input on three frames. It performs well on the Near Collision test set [25] as it was trained from the same dataset but is also able to generalize well on pedestrian collisions on a dataset with similar context but different locations. It is, however, unable to predict well for different kinds of obstacles as the shape and relative speed may differ too much from the data originally trained on (incoming walking pedestrians). Restricting the input data to the obstacle spatial coordinates instead of the input video frames enables better training for the Time-to-Collision task.

The proposed method shows that obstacle Time-to-Collision forecasting on monocular smartglasses can be addressed while restricting the context scenario to specific situations. As we could generalize the task for a pedestrian over a different dataset, the results on static obstacles suggest that a different training of the same network specifically on static obstacles could also generalize to different context datasets. The model cannot predict an accurate estimation of a person moving away from the user as in [25] because it is not trained with this event in mind. The Time-to-Collision forecasting horizon would have to be over the six seconds limit we have set.

Future work may add a tracker and propose multiple obstacles Time-to-Collision forecasting. This task reduced as a regression problem can easily be generalized to different environments.

## Figures and Tables

**Table 1:** Distribution of absolute error (mean ± std) on the Near-Collision test set using different values for the number *M* of hidden layers and the number *K* of neurons per hidden layer with *N* = 6 input frames.

*K* Neurons\*M* Layers	2	4	6	8	10
10	0.383 ± 0.440	0.387 ± 0.414	0.334 ± 0.381	1.140 ± 0.796	1.135 ± 0.816
20	0.361 ± 0.395	0.368 ± 0.419	0.394 ± 0.425	0.332 ± 0.384	1.140 ± 0.797
30	0.358 ± 0.410	0.366 ± 0.417	0.335 ± 0.395	0.338 ± 0.390	1.138 ± 0.803
40	0.361 ± 0.413	0.368 ± 0.416	0.334 ± 0.385	0.334 ± 0.396	1.140 ± 0.797
50	0.339 ± 0.383	0.365 ± 0.405	**0.324 ± 0.383**	0.340 ± 0.400	0.352 ± 0.414
60	0.353 ± 0.405	0.359 ± 0.405	0.339 ± 0.394	**0.320 ± 0.375**	0.328 ± 0.395
70	**0.333 ± 0.374**	0.354 ± 0.422	0.339 ± 0.396	0.323 ± 0.389	0.333 ± 0.399
80	0.341 ± 0.388	0.356 ± 0.418	0.350 ± 0.408	0.324 ± 0.391	**0.321 ± 0.386**
90	0.334 ± 0.373	**0.340 ± 0.408**	0.334 ± 0.395	0.340 ± 0.407	0.335 ± 0.406
100	0.367 ± 0.404	0.362 ± 0.407	0.344 ± 0.408	0.326 ± 0.387	0.324 ± 0.387

Results in bold are the best per number of hidden layers M.

**Table 2:** Distribution of absolute error using different numbers of frames with *M* = 8, *K* = 60.

Number of Frames	Mean (s)	Std (s)
2	0.350	0.435
3	0.327	0.389
4	0.321	0.376
5	0.332	0.398
6	0.320	0.375

**Table 3:** Distribution of absolute error (mean ± std) for Near-Collision network with different input types on the Near-Collision dataset.

Input Data	Mean (in s)	Std (in s)
RGB	0.846	0.666
Masked RGB	0.672	0.567
SimpleTTC (Ours)	0.320	0.375

## Data Availability

Our generated dataset for testing is not publicly available. The NearCollision [25] dataset and code is available at https://aashi7.github.io/NearCollision.html (accessed on 15 February 2021).
